# Exploring vision transformers for deep feature extraction and classification in video genre recognition for digital media

**DOI:** 10.1038/s41598-026-45087-y

**Published:** 2026-03-22

**Authors:** Fawaz Khaled Alarfaj, Anam Naz

**Affiliations:** 1 Department of Management Information Systems School of Business, King Faisal University, Al Ahsa, Saudi Arabia; 2 Department of Information Technology, University of Sargodha, Punjab, Pakistan

**Keywords:** Deep learning, Computer vision, Multimedia analysis, Feature extraction, Vision transformers, Waveform, Acoustics analysis, Management information system, Computer science, Computational science

## Abstract

The generation of video content for television production using automation and artificial intelligence-based techniques is quite common these days. The use of computer vision techniques plays a significant role in the classification and analysis of large volumes of multimedia content. This study aims to develop an intelligent framework for TV genre classification using deep learning and advanced transformer-based models. Traditional machine learning depends on traditional features and lacks the ability to capture complex spatio-temporal and acoustic relationships in modern media. To address these limitations, the study explores state-of-the-art vision transformers for deeper analysis on two standard datasets in the relevant domain. Firstly, a static image dataset is analyzed using the Pyramid Vision Transformer (PvT), which effectively captures multi-scale spatial and contextual information across diverse TV scenes. Secondly, a multimodal audio–video dataset is used by applying the Multimodal Attention and Invariant Vision–Audio Representation Transformer (MAiVAR-T). The applied model captures temporal dependencies and integrates acoustic features, including mel-spectrogram, chroma, waveform, and energy patterns. Empirical analysis demonstrates that the proposed PvT and MAiVAR-T models achieve the highest accuracies of 97% and 98%, respectively, outperforming the baseline deep learning models. This study presents the role of multimodal transformers in improving automated genre classification in television and digital media production.

## Introduction

**Fig. 1:**
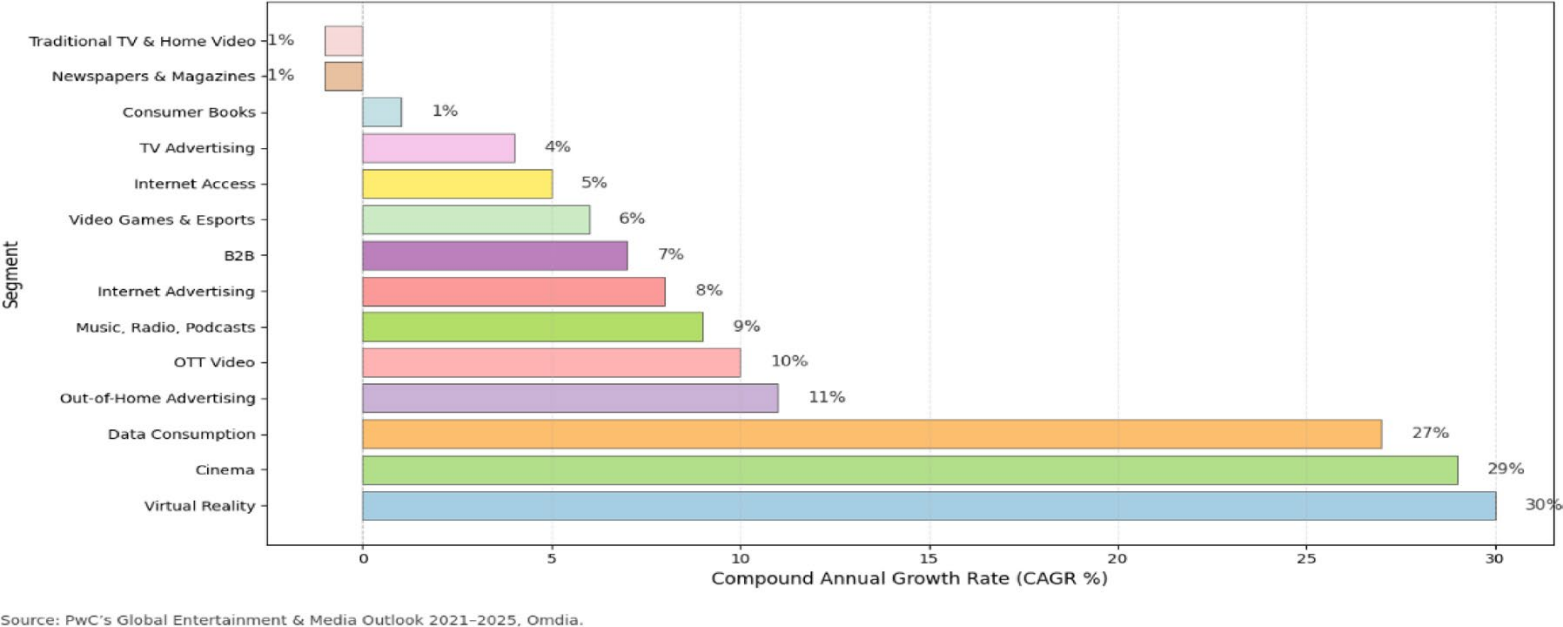
Projected annual growth rates (%) of key entertainment and media segments (2021–2025), highlighting OTT video and traditional television trends.

The use of artificial intelligence plays a significant role in modern television and digital media production. It utilizes visuals, sound, and motion to analyze content across different genres such as drama, comedy, and documentary^1^. Visual analysis plays a key role in understanding and organizing this content, helping producers, editors, and broadcasters manage large volumes of video material^2^. The television and media production market has recorded consistent global growth in the past few years, particularly in streaming content across audio, video^3^, and digital platforms^4^. The world television industry is projected to reach USD 550.8 billion in 2032, up from USD 366.3 billion in 2024, with a compound annual growth rate (CAGR) of 5.23%^5^. In the meantime, the broadcasting and cable TV segments, which still form the core of media infrastructure, were estimated at USD 356.45 billion in 2024 and are projected to increase to approximately USD 449.91 billion in 2030 with growth estimated at around 4%/yr^6^. Investment in content has proved strong despite 2023; worldwide content spending in 2024 is projected to grow by 2%, reaching USD 247 billion^7^. As shown in Fig. 1 (adopted from^8^, these trends indicate that television production, whether traditional broadcasting or streaming, is a powerful and growing industry^9^. As the demand for original content grows, diversity in genres (drama, comedy, reality), and delivery over digital media, the importance of automated genre classification (through computer vision) gains importance to media corporations and television producers in need of a scalable content management system and optimization^10^.

In today’s fast-growing media industry, computer vision and artificial intelligence have enabled the automatic identification and classification of video genres^11^, which can support many areas such as content recommendation^12^, scene retrieval^13^, and production planning^14^. Recognizing several types of television content, such as news, plays, or entertainment, helps improve both creative storytelling and audience experience^15^. Traditional methods for video genre classification depend on conventional features and rule-based algorithms. These techniques tended to address low-level image features such as color, texture, and movement but were unable to capture higher-level visual meaning^16^. Consequently, they did not work well with complex and dynamic television content in which scenes, lighting, and emotions shift quickly. Machine learning and deep learning provide more effective mechanisms for extracting meaningful visual and audio patterns, offering greater accuracy and flexibility for analyzing TV and digital media in real-world situations^17,18^.

This study aimed to cover three types of analysis using publicly available datasets. The first focused on image-based genre classification with four classes (Action, Animation, Romance, and Horror). The second explored multimodal analysis, combining audio and video data with eleven genres to understand scene transitions, sound cues, and emotional tone. The third evaluated model interpretability using Grad-CAM and LIME to explain the decision-making process of the model. The proposed vision-based MAiVAR-T model was designed to manage complex relationships between image, audio, and video features, achieving high accuracy and interpretability.

In this study, the main research contributions include:

- Development of a vision-based multimodal transformer model (MAiVAR-T) that integrates both visual and audio features and pyramid vision transformers for image-based analysis used for improved genre recognition at an accuracy of 98% and 97%, respectively.
- Introduction of a dual-stream framework combining key-frame selection for video and Mel-spectral, Chromogram, MFCC, waveforms, with energy intensity feature extraction for audio.
- Application of explainable AI, including Grad-CAM and LIME, to visualize model decisions and ensure transparency in classification outcomes.
- Evaluation on two diverse datasets, static image and multimodal audio-video, showing superior performance over traditional and deep learning based standard methods.

**Fig. 2:**
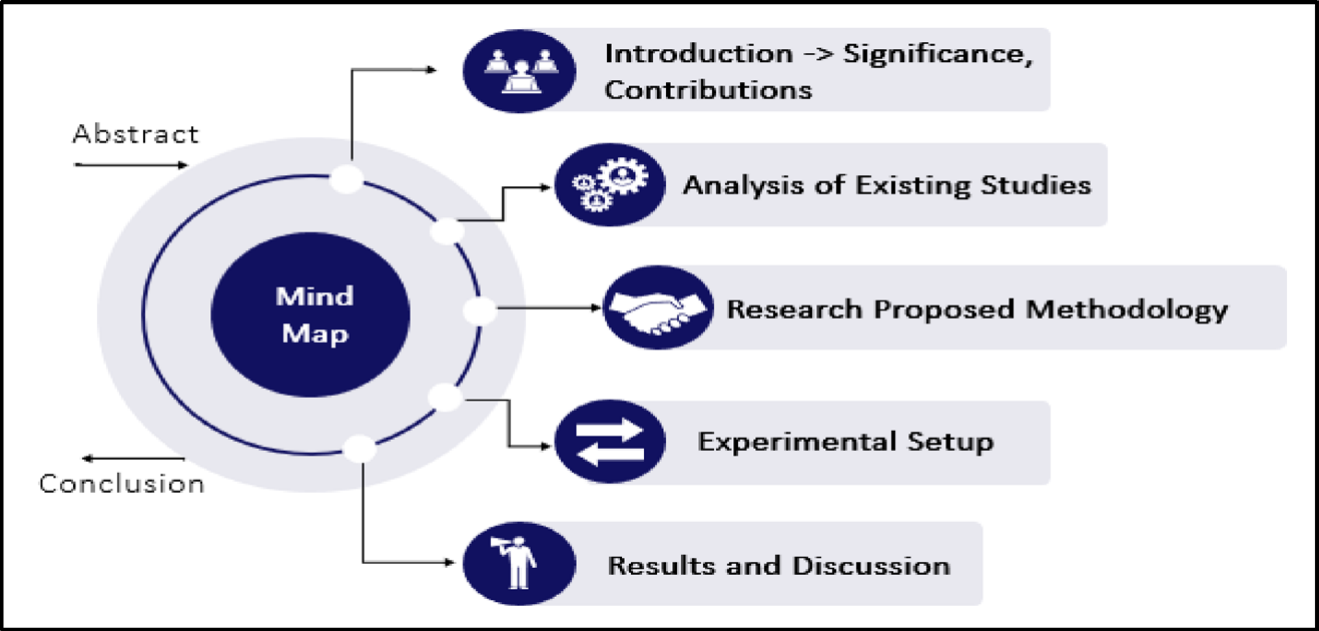
Structural diagram of paper organization.

This study is organized logically, as shown in Fig. 2. The introduction presents the significance of multimedia genre recognition and the research problem. The work section relates to and discusses previous studies and where the multimodal analysis can be taken further. The methodology section outlines the research proposal MAiVAR-T framework, data, preprocessing, and model training procedure. There are findings, visual interpretations, and comparative evaluations in the results and discussion section. In conclusion, the principal findings are summarized, and the importance of the research to the study of automated video genre classification in television and digital media production is also highlighted.

## Related work

The classification of video genres has improved over the past years through the implementation of deep learning models that capture high-quality visual features and model temporal patterns^19^. Vision Transformers have demonstrated strong adaptability across numerous detection and recognition tasks, including audio-visual speech synthesis using transformer-enhanced autoencoders^20^, plant disease classification through optimized transformer architectures^21^, and medical diagnosis, such as melanoma skin cancer detection using deep feature embeddings^22^. These advancements highlight the versatility and effectiveness of transformer-based models in extracting rich spatial–temporal representations across diverse domains. In the early days, working with multi-genre content was difficult, but nowadays CNNs, RNNs, and transformers are used to enhance accuracy^23^. Candela et al.^24^ proposed a comparative deep learning architecture that classifies television shows, with one pipeline applying structural similarity pointers using a CNN on stacked frames and another applying optical flow using a transformer. The former method was effective in identifying program start-end segments and content, with an accuracy of approximately 85%, whereas the latter method examined motion using a shot-boundary module and a transformer, achieving 77% accuracy for multi-class genre classification of the programs. The other tendency is the exploitation of the trained deep models on the multimodal data. Sulun et al.^25^ created a genre classifier that combines an assortment of pretrained feature extractors, which are visual, textual, and audio-based feature extractors. Their technique runs all frames and audio from movie trailers through a transformer that does not spatially downsample the data, enabling intricate correspondences between scenes, objects, speech, music, etc. Likewise, Shao et al.^26^ proposed an ensemble network with a GRU that combines spatial CNN features and temporal motion features to identify a movie’s genre. Their CNN + ensGRU hybrid network was able to capture both the appearance and changing scene context, achieving an F1-score of 0.91 and an estimated accuracy of 94.4% on the LMTD trailer dataset, which was much better than more traditional single-stream models. Lin et al.^27^ also integrated an EfficientNet-B7 CNN to extract frame features and a Bi-LSTM to train temporal sequences to achieve movie genre tagging on clouds with 93.5% accuracy. These recurring network-based approaches demonstrate that combining a deep CNN encoder with an RNN (GRU/LSTM) is effective for leveraging spatiotemporal information to classify long video sequences, unlike frame-based classifiers.

In addition to a simple CNN-RNN stack, researchers have considered the more complex constructions and attention. The use of transformer-based models in video genre recognition is becoming more common. Zhang et al.^28^ introduced a new genre classification method, Movie-CLIP, which augments images with language information derived through video audio (ASR transcripts) and uses a shot sampling method, which eliminates redundant frames. With their model, they were able to boost generating genre predictions mAP by 6–9% on MovieNet and Condensed Movies benchmarks over baselines by a few representative shots, including a dialog/narration cue. It shows that the narrative context (e.g., voice-over or dialogue marks that there will be a comedic tone) can be of significant help to the visual classifiers - a phenomenon that also occurs in hybrid text-video models. Indicatively, Shaukat et al.^29^ were concerned with textual plots through the application of a Genre Attention Model (GAM) that uses a hierarchical attention on transformer-based language embeddings. The deBERTa two-tier attention, which can be interpreted, got a micro-precision of approximately 83.3% on multi-label genre datasets (Trailers12k, LMTD-9, Movie Lens), comparable to visual methods. Recent transformer models for video have also had an indirect effect on genre recognition. Khan et al.^30^ presented MetaBERT4Rec, for action recognizers based on pure vision transformers, and demonstrated that large-scale attention networks can be good at video tasks. Similarly, Moreno-Galvan et al.^31^ predicted the genre and emotion of the viewer using the Bi-Projection Multimodal Transformer (BPMulT) that dynamically combined video and audio streams on an auxiliary emotion recognition task. Their method of rearranging and integrating modality-specific projections enhanced the overall classification accuracy. Multi-label classification and data scale have also been primary factors, alongside architectural innovations. According to Unal et al.^32^, most movies belong to more than one genre, and the genre proportions are highly skewed, making them challenging to learn. They dealt with this by training and comparing six state-of-the-art CNNs (VGG16, ResNet, DenseNet, InceptionV3, MobileNet, ConvNeXt) on a large multi-label poster dataset. Their research concentrated on the case of static posters, but they concluded that transfer learning using CNNs (or more specifically ConvNeXt) is much more effective than training. Temporal indicators using an audio-based genre classifier by Pham et al.^33^ took L3-net embeddings of complete broadcast programs and achieved almost 82% accuracy on BBC TV genres, which was better than previous short-segment audio models. Multimodal fusion is still one of the prevailing ones. One of the initial deep audio-visual genre classifiers was designed by Ben-Ahmed et al.^34^, who used a ResNet-152 to extract the visual features and Sound Net to extract the audio ones prior to fusing the two to predict the genre and the interest of the viewer. Their hybrid model gave a high precision (~ 90%) and recall (~ 87%) on the PMIT dataset, an impressive improvement at the time, but only with mid-level fusion. In subsequent work, Braz et al.^35^ used a dual-stream network to combine poster images and plot text, and suggested the Gated Multimodal Unit (GMU) to adaptively combine text and image features. The model based on GMU achieved an F-score of approximately 0.63 on the 23-genre MM-IMDB dataset, achieving better results than modalities. Conversely, Chu et al.^36^ concentrated on movie posters and demonstrated that a fine-tuned deep network (ResNet-34) was able to categorize a small set (5) of genres with a high quality (around 90.6%).

In the case of movie trailers, the frame and clip-based encoders combined with a fusion transformer in a dual image video transformer perform better compared to single-stream CNN baselines. It is revealed in the study that transformer attention across temporal scales improves genre prediction compared to traditional CNNs, particularly on long trailers. It offers a useful set of recommendations when substituting CNN backbones with transformer blocks like CaiT in the case of a significant temporal diversity^37^. Furthermore, a CNN image encoder (Inception-V4) is used along with a Bi-LSTM temporal head to classify movie genres based on a video clip. The design demonstrates that the spatial features are insufficient in their ability to enhance accuracy on the trailer datasets, but sequence modeling has a significant beneficial impact on improving the accuracy. This work provided a CNN baseline on video and inspired more transformer variations with larger temporal context^38^. The recent developments in multimodal sentiment and sarcasm identification indicate the increasing role of considering visual, textual, and acoustic information for a more contextual apprehension of emotion^39^. VyAnG-Net is a new multimodal model, which is useful in integrating visual, acoustic, and glossary features using a depth-attention component and multi-headed feature fusion to provide sarcasm detection with greater adaptability on different datasets^40^. Likewise, another method also exploits cross-modal semantic incongruities between image, caption, and text through attention to detect sarcastic mood in multimodal data like memes, which outperform previous baselines^41^. Visual-to-Emotional-Caption translation network in sentiment analysis is used to bridge the facial emotion expression and caption attributes of sentiment recognition by providing more aspects^42^. Elaborating on the idea, a contrastive learning-based multimodal architecture takes advantage of image-text pairs to predict emoticons and maps the two modalities to a common latent space to generate more accurate and generalized results^43^. The overall survey further summarizes these progresses with the point that deep learning-based fusion of visual, audio, and text modalities^44^ has enhanced the accuracy and flexibility of multimodal sentiment analysis, and challenges of cross-domain generalization and interpretability remain^45^.

In general, the video detecting genre improves strongly using deeper features and classification methods. Models have advanced to being more complex ensembles that consider spatio-temporal visual cues, audio cues, and even language context. Deep learning has made the content of movies, TV, and online videos much richer to understand, not by applying crude metadata-based tagging but by analyzing the visual story, the sound mood, and the voice-based context provided^46^. Multimodal transformers with large-scale training are getting close to human-level classification and are being used in real-world media services to automatically organize, recommend, and filter content based on these methods^47^.

## Research proposed methodology

**Fig. 3:**
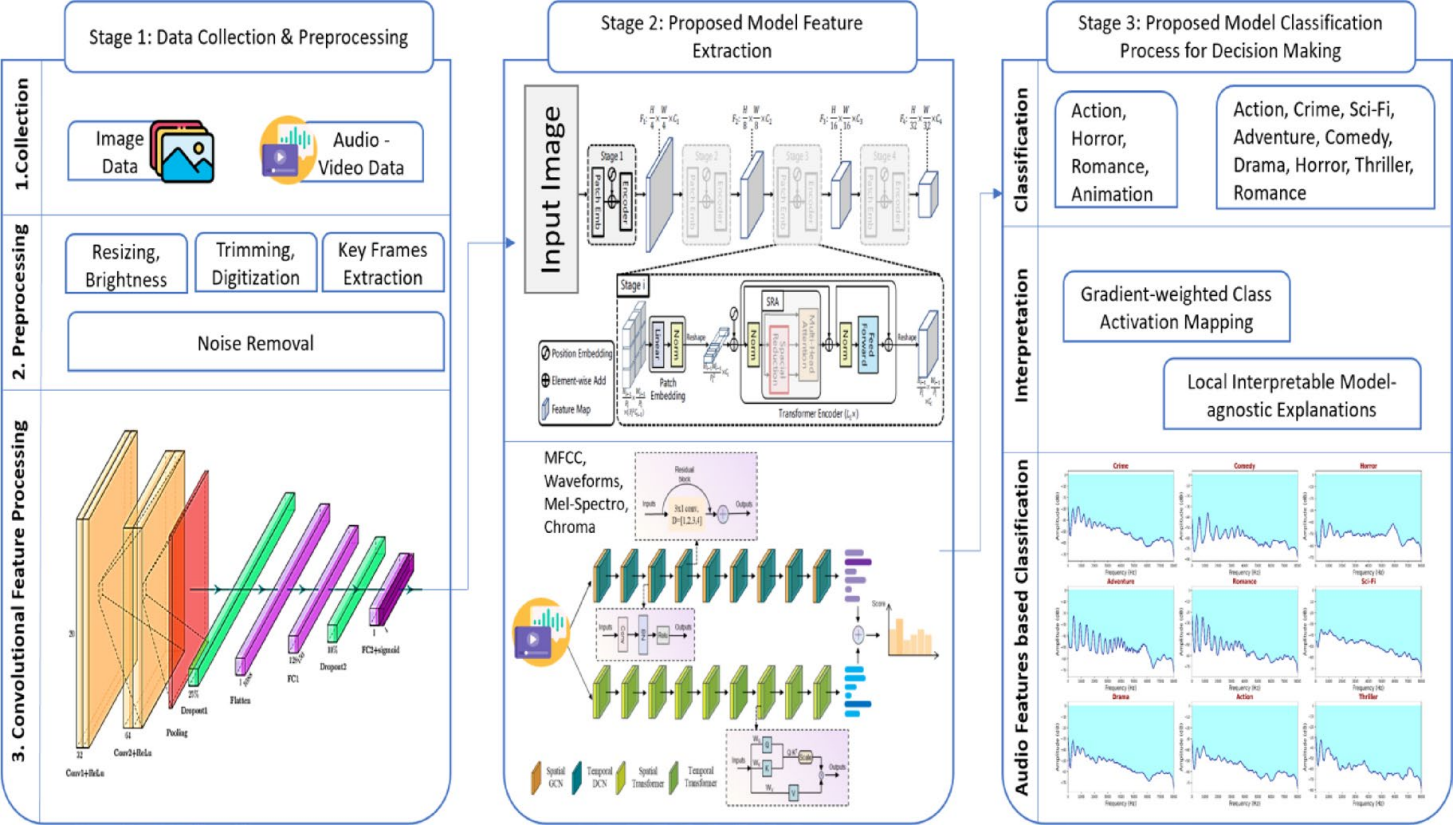
Analysis of proposed framework using research methodology.

The section provides a discussion of proposed methods used in designing, training, and evaluating the proposed models used in video genre recognition, a framework shown in Fig. 3. The data collection and preprocessing, feature extraction, classification, and comparative evaluation are parts of the methodology based on the baseline deep learning models. All the steps were well-planned, so that quality of inputs, the best training, and evaluation were conducted in a regular way.

### Data collection and preprocessing

This study adheres to established ethical research practices. All datasets used are publicly available and anonymized, ensuring that no personal or sensitive information is disclosed. The research does not involve human subjects directly, and no content was collected through invasive or unauthorized means. For static image analysis, a dataset is collected from the online platform Roboflow based on TV production classes. With a total of 4,475 images depicting the scenes of television production, it encompassed four different genres: Action, Animation, Romance, and Horror. The dataset is diverse and balanced in terms of indoor and outdoor visual context, production shots, and varying light conditions are represented. The images were preprocessed to improve the quality and homogeneity of the images prior to model training. The elimination of noise was done, all images were resized to a constant size of 224 * 224 pixels to ensure model consistency in input, and the brightness was normalized to adjust the differences in illumination. The generalization of the model and overfitting prevention were also achieved through additional augmentation, like horizontal flipping and random rotation^48^. The processed dataset was further broken down into training (70%), validation (15%), and testing (15) data. The problem of generalization is due to only a small number of genres, and the model is hard to be effective on unseen or mixed-genre visuals. Data bias occurs due to some of the genres that are prevalent in the training, so that the model will lean towards a particular visual or thematic pattern. Expanding genre diversity to attain balanced and unbiased learning.

For multi-modal analysis, LMTD-9 data was used, which is a large set of movie and television trailers of eleven different genres: Action, Adventure, Animation, Comedy, Crime, Drama, Horror, Romance, Sci-Fi, Thriller, and Documentary. All the samples consist of video and audio, which can be synchronized, so that their visual and audio signals can be processed simultaneously. The preprocessing phase entailed the extraction of key frames of video clips to minimize redundancy and concentrate on the most informative frames^49^. The key frames were chosen according to the change of the scene and intensity of motion, but the moments that could be visually differentiated (e.g., the action scenes, emotional close-ups, or dialogue sequences) were kept. Frame sequences of each video were manually divided at a fixed frame rate, and sample key frames were resized to 224 * 224 pixels to have equally sized sample inputs. Immediately, audio files were ripped and saved in WAV format and equalized to 44.1 kHz. Preprocessing pipeline in audio was noise reduction, amplitude normalization, and trimming silence to enhance clarity prior to calculating features.

### Feature extractions

The Pyramid Vision Transformer (PvT) is applied for extracting features from images due to its capability to extract local spatial content and global contextual information, and is therefore suitable especially in visual understanding in TV production scenes. PvT uses multi-head self-attention and hierarchical token embeddings, unlike the traditional CNNs that use the local patterns by convolution to learn the complex inter-relationships among pixels and objects in the image. In this case, the processing of every image by the PvT model follows four levels^50^. Table 1 shows the layer-wise working of PvT model in this scenario. The embedding layer initially slices the picture into patches, which are linearly projected into tokens. Then the tokens are fed into transformer encoder layers that have several attention heads. The MLP block increases and enriches the feature representations, and normalization layers stabilize the training. Lastly, a connected classification head is then used to produce the genre prediction.

**Table 1:** Pyramid Vision Transformer (PvT) layer configuration and function.

Layer name	Configuration	Function
Patch embedding layer	224 × 224 patch size, stride 4	Converts image patches into token embeddings
Transformer encoder block 1	64-dim embedding, one attention head	Extracts low-level spatial features
Transformer encoder block 2	128-dim embedding, two attention heads	Captures texture and motion cues in production scenes
Transformer encoder block 3	320-dim embedding, five attention heads	Learns global scene context and genre-specific semantics
Transformer encoder block 4	512-dim embedding, eight attention heads	Aggregates multi-scale features for classification
MLP layer	256–1024 neurons (GELU activation)	Nonlinear feature refinement and dimensionality reduction
Classification head	SoftMax output (4 classes)	Predicts genre: Action, Animation, Romance, or Horror

Furthermore, Audio cues give necessary messages concerning mood, rhythm, and transition of scenes in television productions. Each soundtrack was represented in four complementary feature representations that operated on the spectral and temporal properties of the audio, including Mel-Frequency Cepstral Coefficients (MFCC), Waveform Energy, Mel-Spectrogram, and Chroma Features, defined in Table 2.

**Table 2:** Audio features extraction and function analysis.

Feature type	Extraction process	Purpose
MFCC	Derived from the long-scaled Mel frequency spectrum using forty coefficients per frame.	Captures human-perceived pitch and timbre, useful for identifying tonal differences between genres such as *Romance* (soft music) and *Action* (loud bursts).
Waveform amplitude	Direct temporal amplitude envelope extracted from raw audio signal.	Represents sound intensity over time; differentiates *Action* or *Horror* scenes with high-energy bursts from calmer genres.
Mel-Spectrogram	Time–frequency representation using 128 Mel bins and twenty-five ms windowing.	Encodes both rhythm and frequency distribution; identifies background scores and ambience variations.
Chroma features	12-bin chroma vectors computed over harmonic pitch content.	Highlights tonal and harmonic progression patterns in *Drama* and *Romance* genres.

The framework for defining multimodal genres was conducted with the help of the MAiVAR-T (Multimodal Attention and Invariant Vision Audio Representation Transformer) model. This structure was created to encode visual and auditory features together using cross-attention and hierarchical fusion. The model works in two main streams, Vision and Audio stream, and then is combined by a Fusion Transformer Block to give a single multimodal embedding. Table 3 shows the layer-wise working of the MAiVAR-T model in this scenario. Visual data (key-frame embeddings) are initially encoded by a PvT, whereas audio data (MFCCs, Mel-Spectrograms, etc.) are transformed into a 1-D convolution and into a temporal stack of transformers. The fusion layer matches the temporal codes of the two modalities with attention weighting, meaning that similar visual and acoustic codes are concurrently processed. Lastly, a classification head generates the label of the predicted genre. This enables us to capture the temporal dynamics of video sequences, the MAiVAR-T model incorporates a temporal attention process in its fusion layer to allow the network to learn the relationship between frames and between adjacent key frames and audio transitions^51^. Temporal masking and attention weighting align sequential key-frame embeddings in such a way that motion continuity and scene progression are maintained^52^. The model also supports multi-label genre representation, which enables the use of features to identify overlapping or mixed genres like action, comedy, or drama because of common shared time and contextual features in its modalities. The strategy of this temporal modeling helps in increasing the generalization capability of the model to complex and heterogeneous television content.

**Table 3:** MAiVAR-T architecture and layer configuration for multimodal data.

Module	Dimension	Function
Vision encoder (PvT)	Four hierarchical transformer blocks (64–512 dim)	Extracts spatial–temporal patterns from key-frames
Audio encoder	1-D CNN (64 filters, kernel = 3) + Transformer (4 heads)	Learns temporal variation in acoustic features
Cross-modal fusion layer	Attention fusion with gating mechanism	Aligns visual and audio embeddings for synchronized understanding
MLP projection head	512 → 256 → 128 neurons	Compresses multimodal representation into compact form
Classification layer	SoftMax (11 classes)	Predict video genres across Action, Comedy, Drama, etc.

The architecture of both proposed models has been explicitly detailed through structured tables, Table 1 for PvT and Table 3 for MAiVAR-T. Each layer is now described in terms of its configuration and the specific functional role it plays in the overall pipeline. For the PvT image-based model, the design explanation clarifies how hierarchical transformer blocks progressively capture low-level textures, mid-level scene cues, and high-level semantic patterns for genre discrimination. Similarly, the MAiVAR-T multimodal model is broken down into its vision encoder, audio encoder, cross-modal fusion layer, and MLP head, showing how spatial–temporal visual features and acoustic patterns are jointly processed. This expanded description ensures transparency in architectural choices and provides a clear justification for how each component contributes to genre classification performance across image and multimodal data.

### Comparative models

To confirm the performance of the suggested PvT model, various deep learning models and pre-trained models are utilized.

*CNN (Convolutional Neural Network)* is a classic deep learning model in the task of image classification. It can extract features using convolutional and pooling layers, which are good at detecting the spatial patterns, but weak at long-range dependencies^53^. *NASNet*, which is a neural architecture search-based model that searches and automatically optimizes network design to extract features. It is much more adaptable but needs a large amount of computing resources^54^. *CaiT (Class-Attention in Image Transformers) is a* new model that has enhanced class-attention layers to enhance representation learning. It works well with large datasets and requires a big amount of memory and time on the GPU^55^. *AlexNet* is a classical CNN architecture having five convolutional layers and three fully connected layers. It offers an early deep learning performance standard but does not have extensive performance in terms of feature generalization on multimodal data^56^. *VGG-19*, a more advanced CNN with uniform convolutions 3 × 3, is known to be able to pick up fine-grained spatial features^57^. *MFST (Multimodal Fusion Spatio-Temporal Network)*, a hybrid (two-stream) CNN–RNN that can be trained together on audio and visual data, which forms a powerful traditional multimodal control^58^. *CMT (Convolutional Multiscale Transformer)* is a transformer-based network that combines feature scale attention in multiple levels to enhance visual context modeling and temporal reasoning^59^.

Comparative analysis shown in Table 4 demonstrated that the PvT and MAiVAR-T architecture is more advanced than all the basic models to capture both the spatial (visual in video and image) and temporal (audio) features of any genre in complex TV production content.

**Table 4:** Comparative summary of model architectures and functional characteristics.

Model	Architecture type	Core mechanism	Input modality	Feature extraction	Computational complexity	Distinct advantage
AlexNet	CNN	5 Conv + 3 FC layers	Image	Low-level spatial patterns	Low	Fast and lightweight baseline
VGG-19	Deep CNN	Uniform 3 × 3 Conv layers	Image	Fine-grained spatial details	Moderate	High accuracy, simple structure
CNN (Custom)	CNN	Sequential Conv-Pooling blocks	Image	Local texture and color features	Low	Baseline for visual genre tasks
NASNet	CNN	Auto-searched cell structures	Image	Optimized hierarchical filters	High	Adaptively designed for efficiency
CaiT	Transformer	Class-attention with deep encoder layers	Image	Global attention and contextual relationships	High	Strong semantic reasoning
PvT	Pyramid Vision Transformer	Hierarchical attention pyramid	Image	Multi-scale spatial and global context	Moderate	Lightweight and scalable visual transformer
MFST	CNN–RNN hybrid	Dual-stream spatio-temporal fusion	Audio + Video	Temporal and spatial pattern fusion	High	Effective for sequence-based multimodal data
CMT	Convolutional multiscale transformer	Hybrid CNN-transformer with multi-scale tokens	Image + Video	Local-global feature integration	High	Better temporal representation learning
MAiVAR-T	Multimodal transformer	Cross-modal fusion of vision and audio streams	Image + Audio + Video	Joint attention on spectral, spatial, and temporal cues	High	Rich multimodal understanding with balanced accuracy–efficiency trade-off

## Experimental setup

This section provides an analysis of the computational environment, software configuration, and model hyperparameters, which are utilized for experimentation. Experiments were conducted under a controlled environment to provide consistency, reproducibility, and optimal model behavior towards image-based and multimodal (audio-video) genre classification.

### Machine and software requirements

The experiments were conducted using a high-performance computing infrastructure, which is set up for deep learning. The software environment and software structure are shown in Table 5. Other programs like Librosa were used in audio feature extraction, OpenCV in frame extraction and preprocessing, and Matplotlib/Seaborn in visualizing the results. The implementations of the models were versioned to ensure uniform execution of the model across training sessions.

**Table 5:** Analysis of machine and software requirements.

Component	Specification
Processor (CPU)	Intel^®^ Core™ i9-13900 K (24 Cores, 3.0 GHz)
Graphics processor (GPU)	NVIDIA RTX A6000 (48 GB VRAM)
RAM	64 GB DDR5
Storage	2 TB NVMe SSD
Operating system	Ubuntu 22.04 LTS (64-bit)
Deep learning framework	PyTorch 2.1.0 + CUDA 12.2
Programming language	Python 3.10
Development environment	Jupyter Notebook/Google Colab Pro
Key python Llibraries	Torch, torchvision, transformers, numpy, opencv-python, librosa, matplotlib, seaborn, scikit-learn, pandas, tqdm

### Hyperparameter settings

To optimize its performance on the multimodal (audio + video) dataset, the Pvt and MAiVAR-T model was optimized for the tuning process, which consisted of optimizing the learning rate, the head configuration of the attention, and the ratios of dropouts to avoid overfitting, but remain stable in convergence. The hyperparameters reported in Table 6 were selected through a combination of prior empirical evidence, standard practices in transformer-based vision and multimodal learning, and controlled pilot experiments on the validation set. The choice of batch size and learning rates was made to balance the stability and limitation of GPU memory, and instead, smaller batches and low learning rates were used in the multimodal MAiVAR-T model, as it represents a more complex model. The epochs, early stopping patience, and cosine annealing scheduler were fixed after noticing that the convergence was not overfitting. Optimizer parameters such as AdamW, β1, β2, *e*, and weight decay are based on transformer settings to provide the proper regularization and smooth optimization. Patch size, embedding dimension, attention heads, and depth were matched to the architectural design of PvT and the needs of fusion in MAiVAR-T, which allowed the learning of multi-scale features at computational efficiency. The dropout and activation mechanisms were adjusted to reduce overfitting and enhance performance on generalization in image-only and multimodal cases.

All compared baseline models, including CNN, NASNetMobile, CaiT, AlexNet, VGG-19, MFST, and CMT, were implemented and evaluated under a unified experimental setup to ensure fair comparison. All the models were trained on the same datasets, with the same training, validation, and test splits. Upon this, ready-trained weights (ImageNet) were employed and adjusted on the target datasets where necessary. The validation set was used to tune the hyperparameters of learning rate, batch size, optimizer, and the number of epochs, and early stopping was utilized to eliminate overfitting. The accuracy, precision, recall, and F1-score performance measurements were calculated in all the models, and the result reported is the best-performing setting of each baseline.

**Table 6:** Hyperparameter analysis of proposed models in both cases.

Parameter	MAiVAR-T (audio + video)	PvT (image-based)	Description
Batch size	32	64	Number of samples processed simultaneously during one training iteration; smaller batch for multimodal stability, larger for image efficiency
Epochs	100	100	Total number of complete passes through the training dataset
Stride	6	4	Step size of convolutional or patch extraction operations, controlling spatial/temporal resolution
Padding	2	4	Zero-padding applied to preserve spatial dimensions during convolution
Epsilon (ε)	1e-8	1e-5	Numerical stability constant in AdamW optimizer to avoid division by zero
Beta 1	0.9	0.99	Exponential decay rate for first-moment (mean) estimates in AdamW
Beta 2	0.999	0.999	Exponential decay rate for second-moment (variance) estimates in AdamW
Dropout in FC Layers	0.3	0.25	Regularization applied to fully connected layers to reduce overfitting
Learning rate	0.0001	0.001	Initial step size for gradient updates; lower rate for multimodal convergence
Optimizer	AdamW	AdamW	Adaptive optimizer with decoupled weight decay for better generalization
Weight decay	0.05	0.005	L2 regularization strength to penalize large weights
Patch size	Key-frames 224 × 224 pixels	224 × 224 patches	Spatial resolution of input frames or patches fed into transformer blocks
Embedding dimension	512 (fused embedding)	320	Dimensionality of token embeddings; higher dimension for multimodal fusion
Number of layers	8 (main + fusion)	4	Depth of transformer architecture; additional layers support cross-modal learning
Attention heads	16 (fusion layer)	[1, 2, 5, 8]	Number of parallel self-attention heads capturing diverse feature subspaces
MLP dimension	512	1024	Hidden dimension of feed-forward networks within transformer blocks
Dropout rate	0.2 (Audio), 0.1 (Vision)	0.1	Modality-specific dropout for balanced regularization
Activation function	GELU	GELU + SoftMax	Non-linear activation; SoftMax used in final classification layer
Learning scheduler	Cosine Annealing	Cosine Annealing	Gradual learning rate decay to improve convergence and stability
Loss function	Cross-Entropy Loss	Cross-Entropy Loss	Objective function for multi-class classification
Patience (early stopping)	5	5	Number of epochs to wait before stopping training if validation does not improve
Pretraining source	Fusion pretraining	ImageNet	Initialization strategy to accelerate convergence and enhance generalization

### Performance evaluation measures

The major classification measures, such as Accuracy, Precision, Recall, and the F1-score, were used to evaluate the performance of the proposed models. The measure of *accuracy* was the total accuracy of the predictions, and *Precision* measured the effectiveness of the model in identifying the true positive samples out of all the predicted positive samples. *Recall* was evaluated as the capacity to identify all the pertinent instances in each genre, and the *F1-score* offered a weighted median of Precision and Recall, gauging the steadiness in classes. The final validation of the results to obtain *statistically* reliable results was to perform p-value analysis under various tests (including t-test, ANOVA, Chi-square, and Z-test), and all obtained a p-value of less than 0.01, which showed that statistically significant improvements were obtained by the proposed MAiVAR-T and PvT models. Also, *Grad-CAM and LIME* were used to investigate the interpretability of the models, and the features and areas most likely to influence every classification decision were highlighted visually. Grad-CAM offered an attention heatmap on top of significant visual regions in frames, and LIME provided insight into the weights of individual predictions, and all ensured the models paid attention to significant attributes (e.g., actor motion, lighting, tonal differences) on genre recognition.

## Results and discussion

Results discuss the entire analysis of the utility of the proposed PvT for static images and MAiVAR-T for multimodal data, including traditional DL and pre-trained models utilized as baseline models for the classification of genre detection.

### **Results with image-based dataset**

Table 7 shows the performance evaluation of the four models, namely CNN, CaiT, NASNet, and PvT, on the classification task of the TV multimedia genre. These models were evaluated on four genres, namely Action, Animation, Romance, and Horror, and their standard classification metrics, such as Accuracy, Precision, Recall, and F1-Score, were used. The traditional CNN model was able to attain an overall accuracy of 89% stable and limited feature extraction ability of intricate visual scenes that are common in TV production content. Although it was able to capture the simple features of space, its relatively lower precision (88) and F1-score (89) indicate that it struggles with capturing delicate features of visual information like subtle visual elements in action-romance hybrids or animated horror sequences, which are found in cinematics. Class-attention-based CaiT model achieved 91% accuracy and showed more genre discrimination based on context-sensitive spatial learning. The fact that it could serve finer frame details made it useful in animation and romance movies, where the expression of characters and the composition of the scene play a more significant semantic role. Nonetheless, medium recall (91) suggests that CaiT sometimes incorrectly categorized cases of boundaries, e.g., scenes of visual darkness based on romance mistakenly labeled as horror, which are sensitive to changes in lighting and tone typical of TV cinematography. The NASNet architecture reached a 93% accuracy, which indicates increased flexibility in feature search and automatic architecture tuning. NASNet, with a recall of 96%, effectively learned dynamic motion in action sequences and stylized patterns in animation and provided a balance in performance in each category. Its weakness is that it can generalize across variable frame rates and production qualities because low-budget or archival content added noise and would therefore decrease precision to a slight degree (93%).

The overall performance was the Pyramid Vision Transformer (PvT) model, which had an accuracy of 95%, 97% precision, and an F1-score of 96%. The hierarchical attention framework of PvT made it possible to learn local and global features in a fine-grained manner, and this supported high differentiation between horror and action genres, which frequently have similar visual tropes, including fast motion and dark color schemes. The recall of the model is 95%, thus showing its strength in processing genre-defining cues despite the production styles. In a real-world analysis of TV production, it implies that PvT could be used in automated content tagging, archival classification systems, and genre-based recommendation systems, which can provide efficient metadata production to streaming services and video broadcasters. The continuous increase in performance of CNN to PvT shows the increasing benefit of transformer-based vision models on complex and highly varying visual data found in TV media. In addition to enhancing the accuracy of genre recognition, these models offer interpretive data of visual storytelling structures, which characterize TV genres, including lighting, framing, and tone. The results apply to the context of the contemporary multimedia production, in which the AI-enhanced classification might optimize the production and processing of editing, cataloging, and audience analytics pipelines to facilitate smoother content management and creative decision-making in the television and digital broadcasting sectors.

**Table 7:** Results analysis using TV production image dataset.

Models	Accuracy	Precision	Recall	F1-score
CNN	89	88	90	89
CaiT	91	93	91	92
NASNet	93	93	96	94
PvT	95	97	95	96

**Fig. 4:**
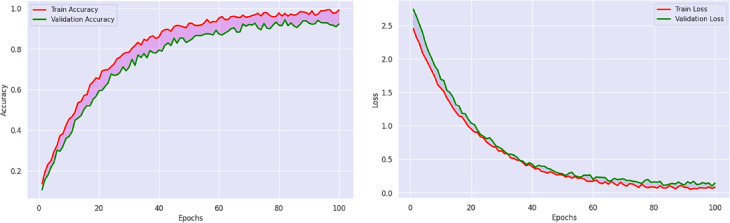
Model results analysis of accuracy over training and validation data.

**Fig. 5:**
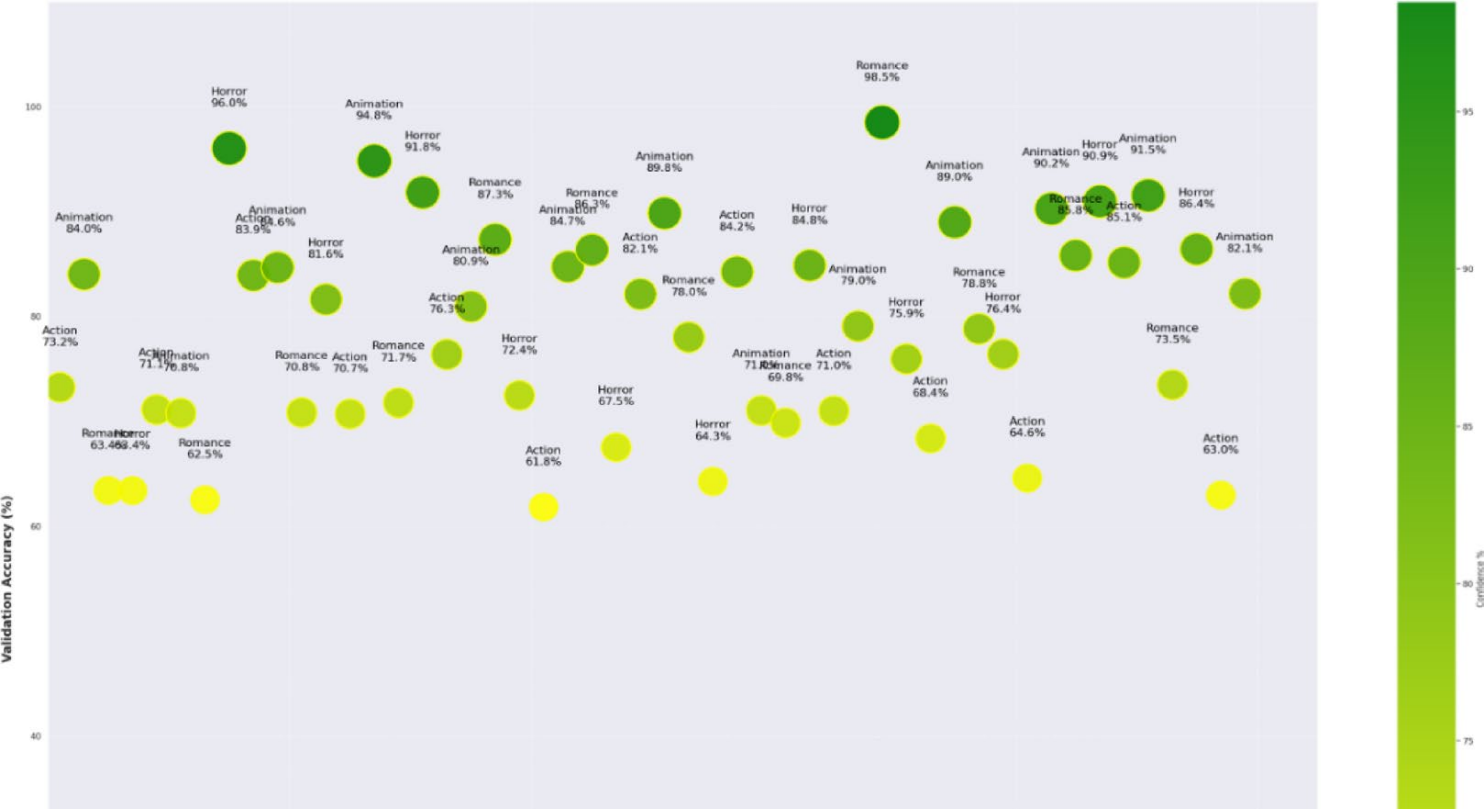
Model confidence results analysis of accuracy based on random sample training.

The plot in Fig. 4 of training-validation accuracy shows that the PvT model gradually improved by gaining accuracy between 95 and 97%, indicating an efficient learning of the features and robust generalization without over-fitting. The resulting loss curves reduce continuously, which is an indication that the model is an effective method to minimize error in the optimization process. This similarity between the training and the validation curve is also indicative of the fact that the model has balanced performance as per the genres, in that it is able to separate those visual patterns that are common to the various categories of television productions, including action, animation, romance, and horror. PvT can clearly capture the long-range dependencies to perceive the genre-specific patterns in scene transitions as well as in the symptoms of lighting and artistic framing. The confidence score accuracy versus random samples in Fig. 5, overall accuracy on predictive outcomes and trends are demonstrated through performance analysis of the PvT model in TV genre classification. It performs well both in feature extraction and pattern recognition, which is shown by the fact that most samples have confidence in more than 95%, and especially in Horror (96.0%), Animation (94.8%), and Romance (98.5). However, some genres show more variance in confidence, which suggests that genres may have characteristic features that would overlap, causing minor mismatches.

The model completes its learning, with an upward trend in the validation accuracy over epochs. The trend line signal staying positive suggests that PvT is generalizing learned patterns from the training set and that it improves the trend line over time. Nevertheless, the fluctuations of accuracy at different epochs are caused by the variability of validation samples, and TV production content is variable in nature, and it is not possible to distinguish between different genres.

### Results with multimodal sata

The results of the comparative analysis of six models, including AlexNet, VGG-19, CNN, MFST, CMT, and MAiVAR-T based on the multimodal dataset, indicate gradual improvement in the accuracy of genre recognition with the increase in the complexity of the architectures and the depth of cross-modal fusion, as shown in Table 8. The data was audio-visual (a combination of audio and visual), there was conversation, background music, and scene dynamics that can be found in eight television genres: thriller, science fiction, romance, horror, drama, crime, comedy, and action.

**Fig. 6:**
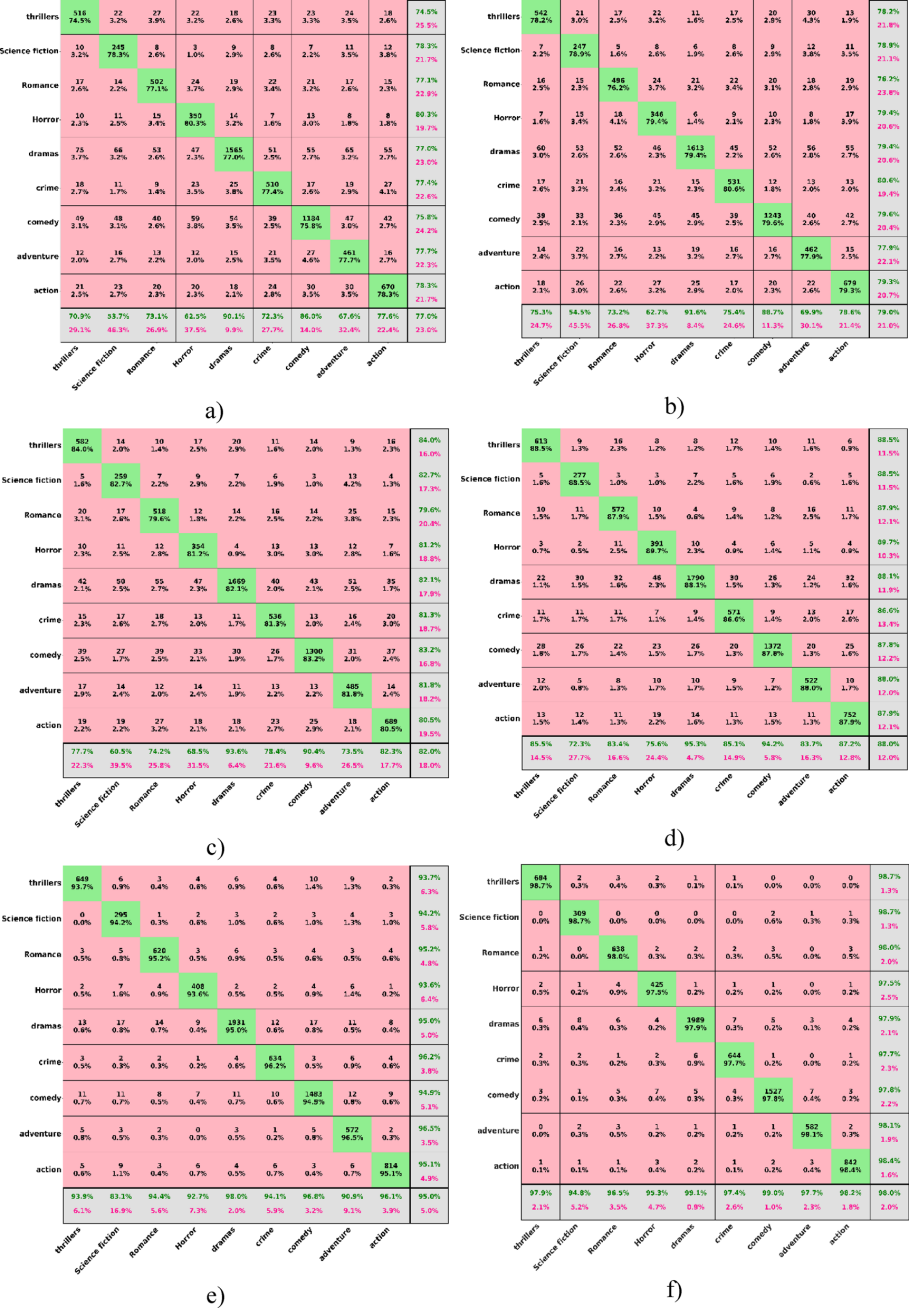
Confusion matrix analysis of all models: (**a**) AlexNet (**b**) VGG-19 (**c**) CNN (**d**) MFST (**e**) CMT (**f**) MAiVAR-T.

AlexNet baseline model was recorded to achieve a general accuracy of 77%, and it serves as a point of reference to early convolutional neural network models that are based on low-level visual features. Its low accuracy and memory (73% and 77%) suggest that it might perceive prominent visual features (high-contrast or action scenes), but was not able to perceive temporal rhythm and acoustic diversity that is important to distinguish between drama and romance. Equally, VGG-19 slightly bettered the accuracy to seventy-nine, which enjoyed the advantages of more convolutional stacks at the expense of multimodal composition. The confusion matrices in Fig. 6 indicate a significant confusion of thrillers and crime genres and romance and drama, underlining the fact that solely visual indicators cannot work when the auditory mood and rhythm determine the perceptions of the genre. AlexNet in Fig. 6a and VGG-19 in Fig. 6b show a misclassification rate of classes of 22%. CNN and MFST models improved their performance as their accuracy was 82% and 88%, respectively. The fact that CNN demonstrated a better recall (82%) implies a better sensitivity to genre-specific textures and motion patterns, but its misclassification, which is apparent in Fig. 6c, remained within sets of visually similar classes, i.e., horror and thriller. The MFST network was an attempt that combined the audio spectrograms with video frame embeddings coordinated, and the results were more balanced across genres. The confusion matrix (Fig. 6d) has a higher diagonal dominance, which is a sign of better temporal context learning. It is important to note that drama and comedy genres have the highest recalls (greater than 88), which is due to blending the speech tone with the features of facial expressions, as it enhanced the semantic fusion between modalities.

**Table 8:** Results analysis using LMTD-9 multimodal dataset.

	Models	Accuracy	Precision	Recall	F1-score
a	AlexNet	77	73	77	74
b	VGG-19	79	74	79	76
c	CNN	82	78	82	79
d	MFST	88	85	88	86
e	CMT	95	93	95	94
f	MAiVAR-T	98	97	98	98

A significant performance of 95%, CMT in Fig. 6e, proved that self-attention is beneficial to understand long-range dependencies between scenes and sound segments, indicating good separation of the genres with recalls of ninety-four and above in all the categories. The fact that the model could deal with arbitrary frame rates and multi-speaker conversations allowed the classification of science fiction and adventure to be much more similar, as both acoustic and visual information are complicated. Yet, there was still a slight overlap between action and thriller, which suggested that high-energy sound and speedy pacing are hard to separate in cases when both films depict the same levels of cinematic intensity. All the baselines were lower in performance with the proposed MAiVAR-T, with a 98% accuracy, a 97% precision, a 98% recall, and an F1-score of 98%. Its confusion matrix in Fig. 6f shows almost perfect diagonal dominance, with each genre having over 97% correct classification.

The attention mechanism of its transformer had two streams that were useful in aligning spectral audio properties (mel-frequency cepstral coefficients and rhythm patterns) with spatio-temporal video embeddings, allowing subtle mood and tone variations to be easily differentiated. As an illustration, the clear separation of romance and drama was the result of the contextual emotional indications of speech prosody and ambient score, whereas the horror and thriller were advantaged by the combined encoding of the audio energy at low frequencies and the light of dark scenes. There are practical implications for this as far as television production is concerned. Proper genre identification refers to automated content classification, highlight mining, and archive search in the media pipelines.

The multimodal description of MAiVAR-T enables the demonstration based on rhythm and acoustic intensity of the work, which is essential in managing and editing workflows. In broadcast management and streaming, this model can simplify the recommendation engine, genre-based scheduling, and rights management by accurately aligning episodes or trailers with genre taxonomies. The confusion-matrix patterns across all models prove that audio-visual fusion can significantly decrease the ambiguity between the genres with similar visual semantics, but unique acoustic features. In comparison to the case of early CNNs, where fusion was performed with no attention movement between the two modalities, transformer-based fusion used both modalities of attention to capture semantic emotion flow and scene continuity. The overall increased performance of AlexNet to MAiVAR-T highlights the shift in the visual perception processes that are handcrafted for multimodal representation learning. High precision, recall, and the F1-score measured by the final model confirm its stability in the practical classification of multimedia, which can be taken as one of the promising steps into the intelligent and production-level genre understanding in TV analytics.

**Fig. 7:**
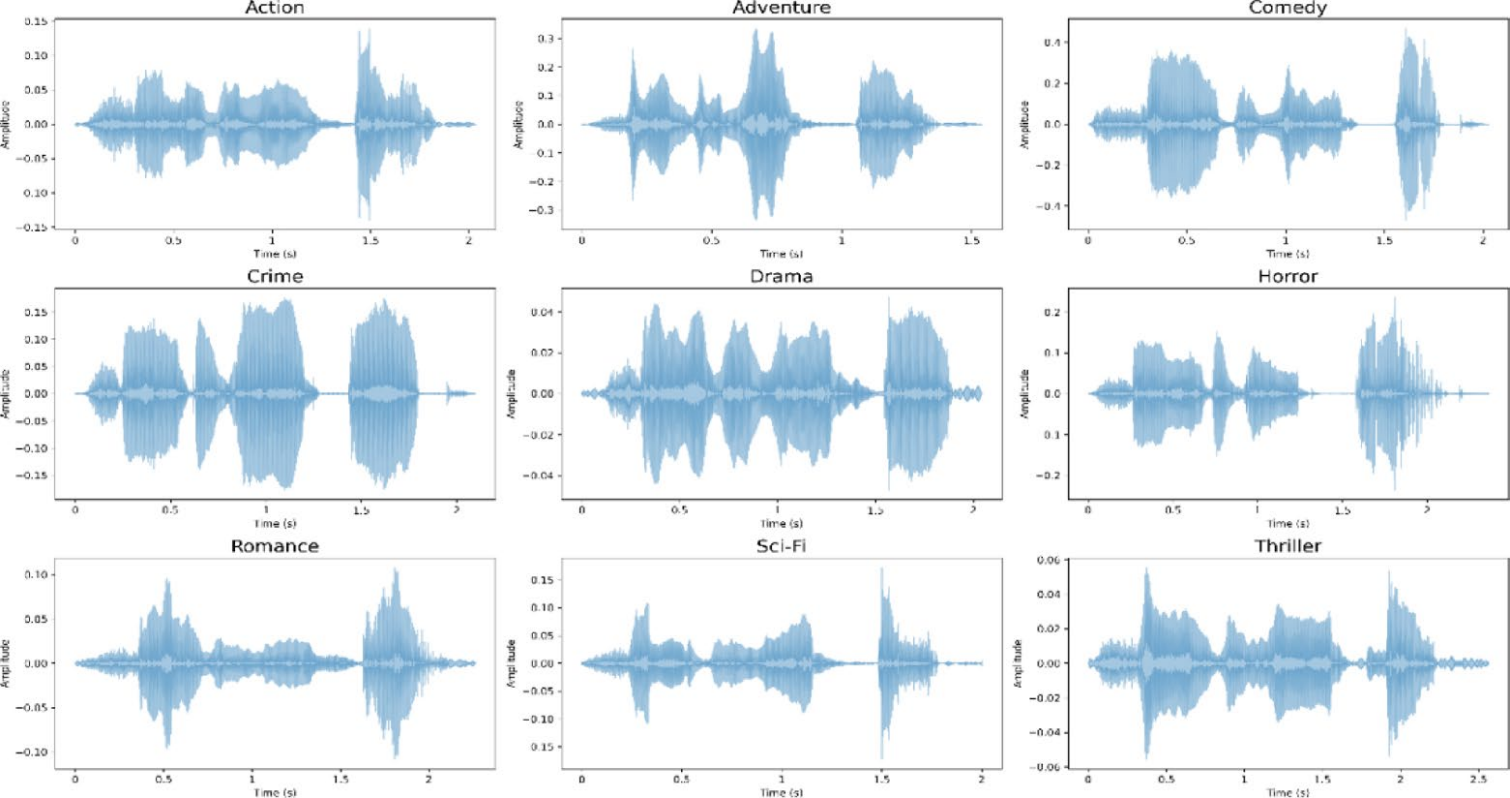
The audio signals waveforms of genre-specific audio signals represent typical patterns of temporal energy and intensity.

The proposed MAiVAR-T model has significant capabilities to identify and decode audio-based features that play a significant role in genre discrimination in the multimodal (audio plus video) system. Figure 7 shows that the waveform patterns have unique temporal and amplitude differences in the genres, and this is due to the tonal energy and rhythmic makeup of each content type. Action and Adventure sequences have an intense peak and short inter-silence moments that indicate thick sonic action like explosions or the quick utterance of words. Romance and Drama genres, in their turn, show less amplitude envelopes and more pauses, which is associated with a softer background music and conversational breaks. This model has been shown to learn these amplitude-temporal distributions, enabling its ability to align the acoustic intensity to the visual tempo of similar frames.

**Fig. 8:**
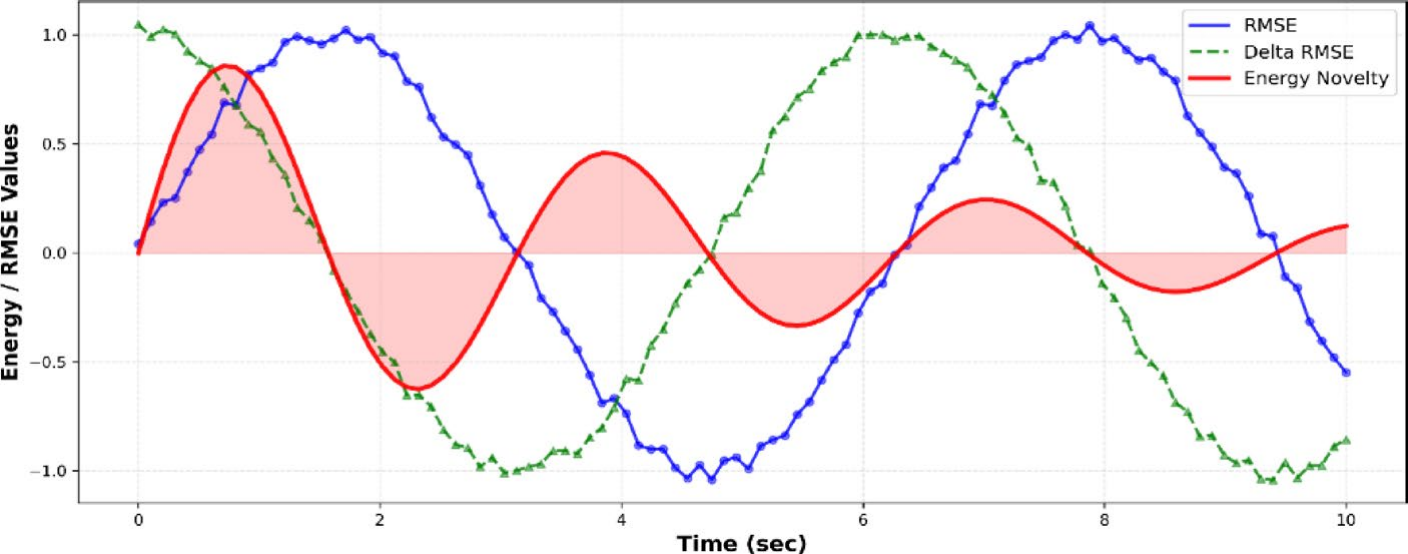
Analysis of RMSE, delta-RMSE, and energy novelty at time showing peaks coincide with scenes of high tension or with a switch in the scene, which facilitates the temporal attention modeling.

The RMSE-Energy Novelty curve in Fig. 8 gives additional details of the dynamic transitions of acoustic trends with time. RMSE measures peak-to-peak swings in the short term, whereas its derivative (DRMSE) indicates time changes in sound levels. The red Energy Novelty curve indicates that the model has been used to recognize emotional transitions in the model, like the accumulation of thriller scenes or the crescendos in melodies in romantic sequences. The attention mechanism of the transformer can capture these fluctuations and place a higher weight on frames over which significant acoustic variations occur, resulting in greater recall of emotion-driven music such as horror and drama. Such a variation of sensitivity indicates the ability of MAiVAR-T to match the emotional rhythm with visual movement, which is a key element of multimedia genre recognition.

**Fig. 9:**
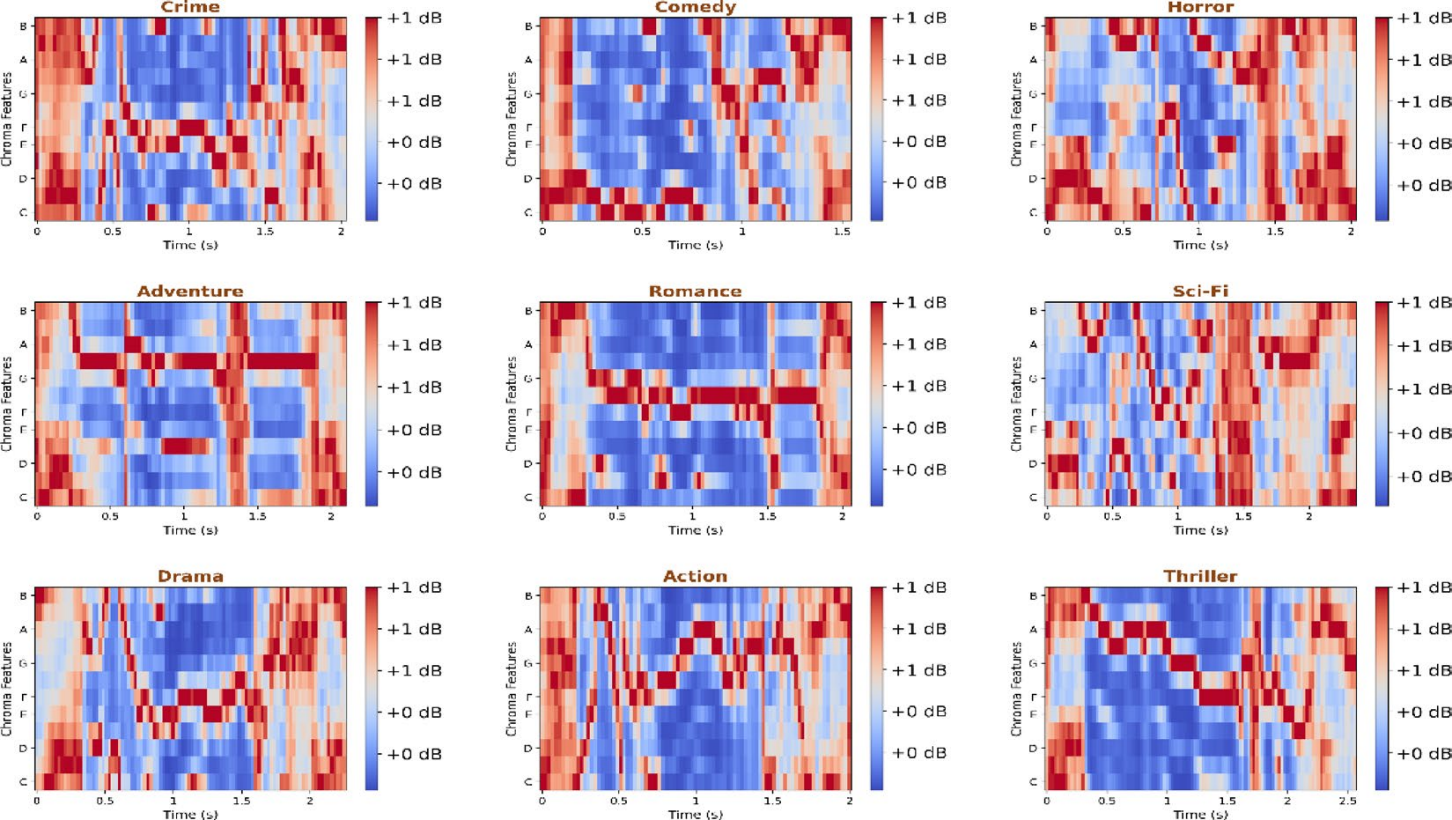
Comparison of tonal and harmonic organization in genres through chromogram distribution highlights genre-dependent tonal stability and pitch transitions.

**Fig. 10:**
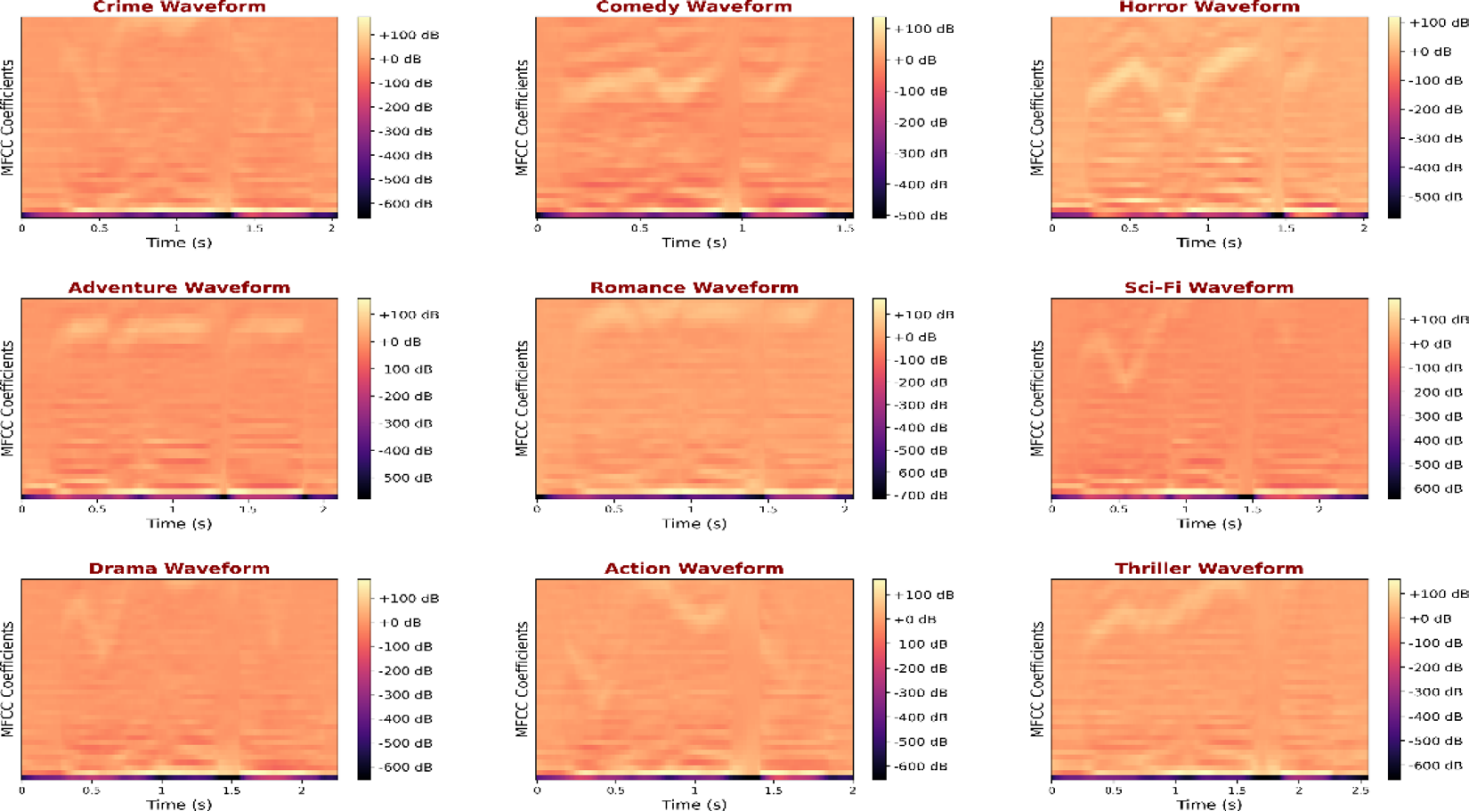
Genre waveforms MFCC heatmap records the spectral envelope and richness of the timbre of a specific genre, showing attainable results of lower and higher frequency dominance, respectively, in the multimodal fusion layer.

Tonal and harmonic distributions presented as chromogram representations in Fig. 9 are used to distinguish musical structures inherent in every genre. To illustrate, romance and comedy demonstrate the stability of the chroma, which suggests the tonal consistency that is characteristic of background melodies. Conversely, crime and thriller genres have irregular chroma energy, which is associated with dissonant sounds and tonal disjunctions. This chromatic diversity is used to the advantage of the model by its cross-attention blocks, as the tonal complexity is mapped onto the intensity of the scene. Such awareness of tone makes it assertive in the identification of thrillers and drama, even though the two may have similar dialogues in their stories, yet they are contrasted in terms of musical tension. The sensitivity of the model to spectral envelopes and timbral nuances is highlighted further by an analysis of MFCCs shown in Fig. 10. The MFCC heatmaps show distinctly concentrated energy of the lower and mid-frequency bands according to the genre. Crime and horror genres exhibit greater lower-frequency content because of the ambient noise and tonal ambience in their sound design, whereas the spectral content of comedy and romance is brighter because of the greater speech and light instrumental components. The frequency distributions are well represented in MAiVAR-T, which incorporates them in its multimodal representation space. The fusion head of the model is MLP-based, which refines these embeddings, increasing inter-genre separability and adding to the near-perfect precision (97%) and recall (98%).

**Fig. 11:**
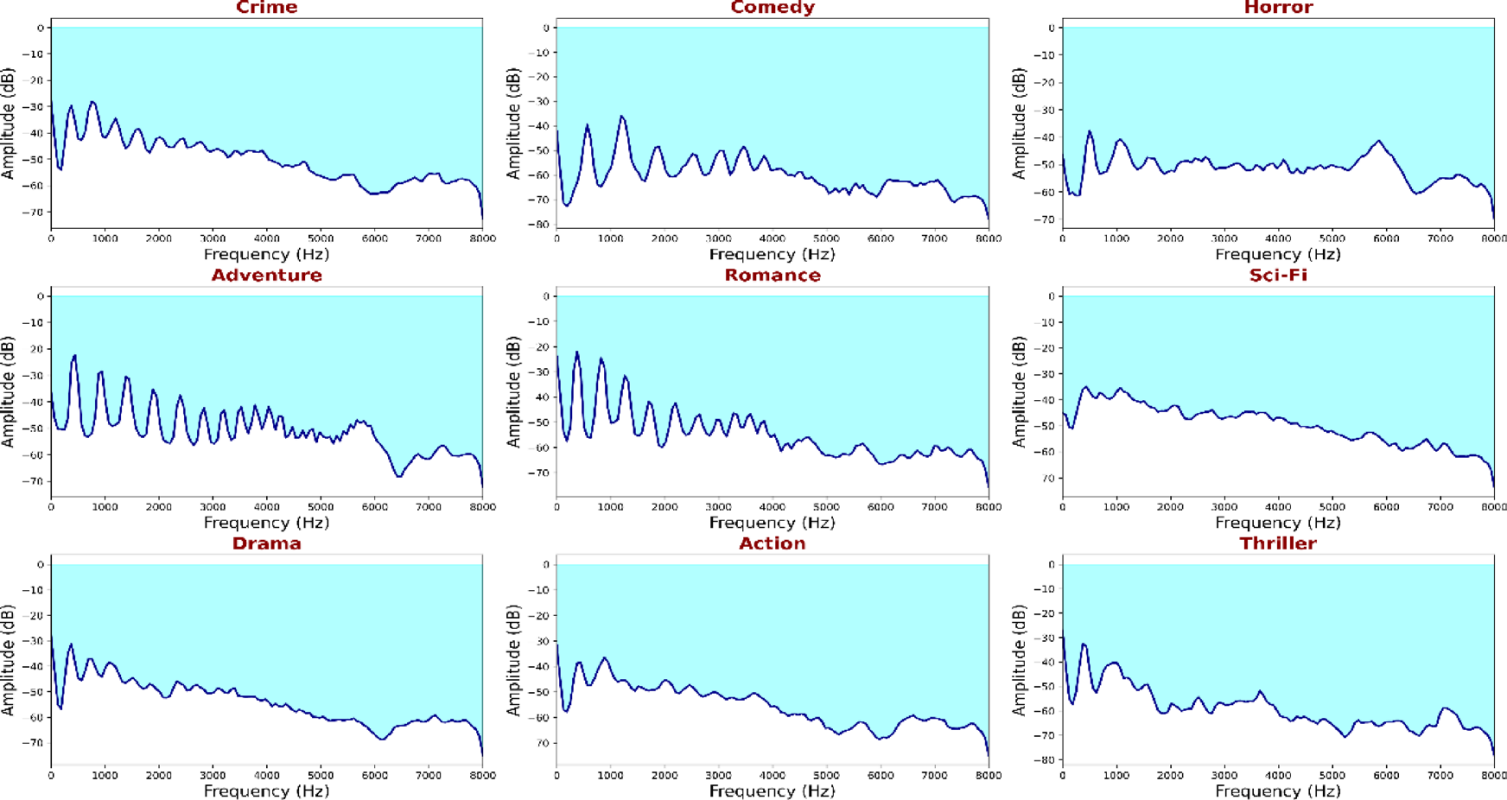
Genre-based Mel-Spectrogram audio representations for tonal and rhythm changes into alignment with the video frame sequence in transformer attention layers, which enhance cross-modal synchronization to make accurate genre classification.

Figure 11 shows an analysis of the Mel spectrograms feature, which demonstrates time-frequency analysis with detailed time-frequency breaks in which the proposed transformer obtains both fine-grained and long-term audio features. The layered attention mechanism detects bursts of energy and harmonic resonances that are specific to the genre - dense high-frequency elements in action and adventure, and low-frequency sustenance in horror and drama. It is a spectral-temporal constructive collaboration, which means that the model can match audio cues with visual events such as explosions, laughter, or dialogue pauses. Combining Mel-spectral and visual embeddings in the MAiVAR-T pipeline will guarantee the stability of genre inference in cases when the visualities are weak, i.e., in darkened or acoustic prevailing images.

These findings confirm that the combination of such features as time, spectrogram, and harmonic in MAiVAR-T provides a full picture of the acoustic environment of TV media and allows proper and context-related genre classification.

**Fig. 12:**
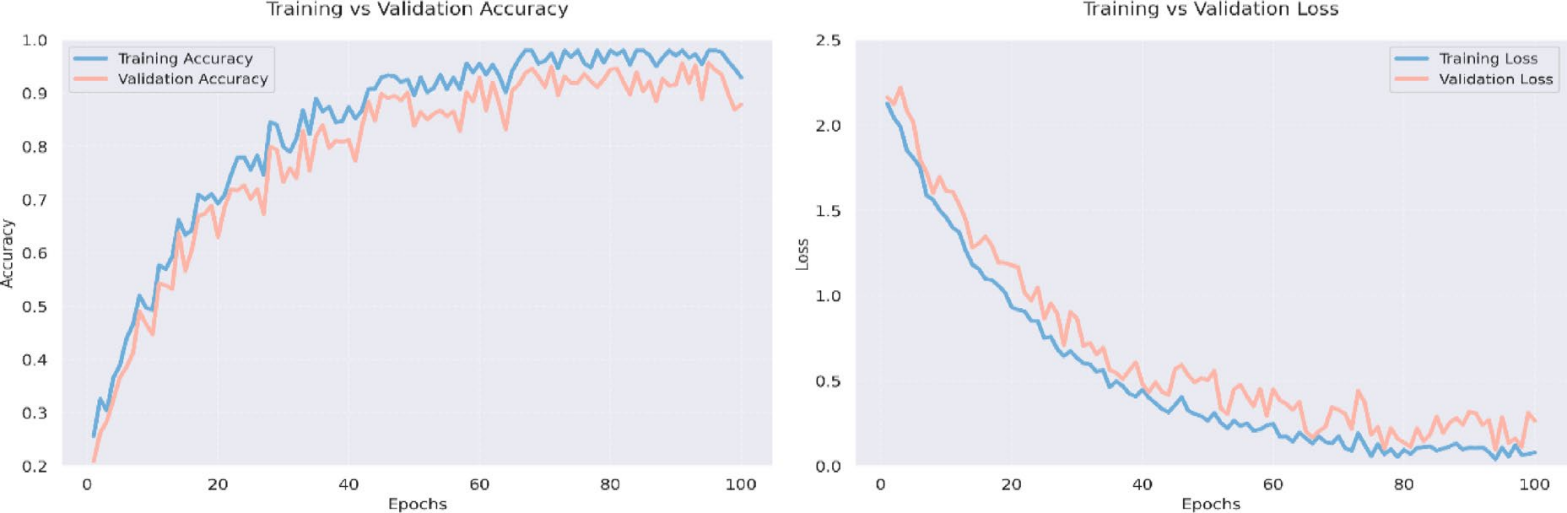
Validity accuracy vs. training vs. loss curves at 100 epochs with stable accuracy increase and smooth loss convergence on training and validation sets, which means a prominent level of generalization and decreases overfitting.

**Fig. 13:**
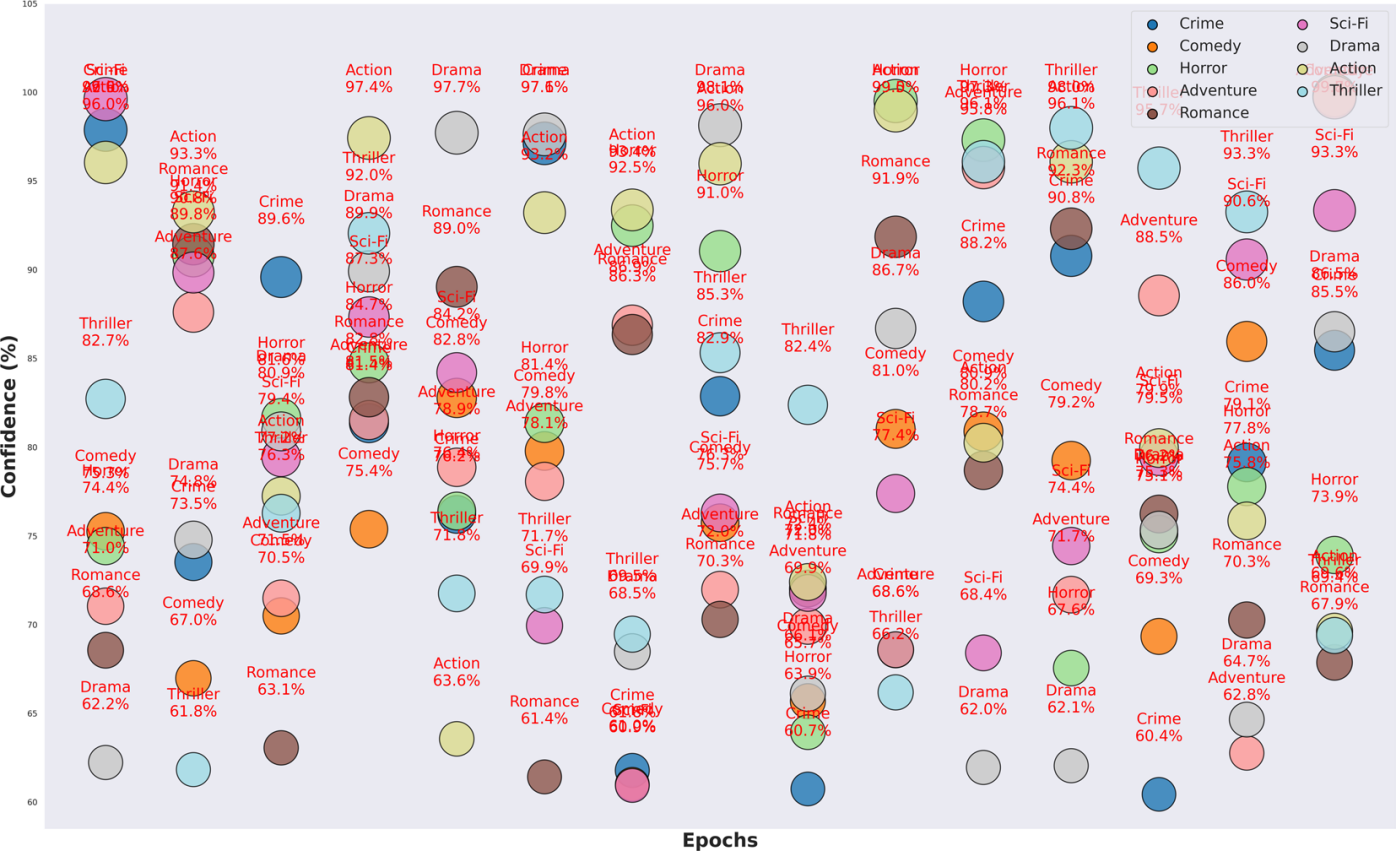
Confidence analysis of the model indicates the magnitude of confidence, which is a feature of consistent stability of genres and equal prediction strength of the multimodal dataset.

Figure 12 shows that the MAiVAR-T model has a high and stable convergence behavior during the training procedure, as indicated by the accuracy and loss curves. Both training and validation accuracy come up steadily, with one hundred epochs reaching performance (98%) with only slight deviation between the two curves. This means that the model does not overfit and is generalized. Accordingly, the training and validation losses decline steadily, reaching a steady point of less than 0.2 after 80 epochs, and this indicates that the optimization process is stable under the AdamW optimizer. The fact that there is a smooth convergence trend confirms the efficiency of the multimodal attention mechanism as well as the selected hyperparameter set to balance cross-modal learning. Figure 13 shows the bubble-based confidence plot, which reveals the genre-specific classification of confidence by epoch. Bubbles are the weights of accuracy of a genre divided by the confidence of prediction. Genres like Drama, Action, Sci-Fi, and Adventure are the ones that continually allow higher levels of confidence (95%), which is indicative of the model’s ability to recognize the genres that feature the presence of strong audio-visual stimuli and movement patterns. The confidence of Comedy and Romance is a bit less, but also confirms the strong presence of this genre (89–91%), probably because acoustic and emotional differences are not so noticeable. The size distribution of the bubbles used also proves that MAiVAR-T does not over-fit on any categories but instead provides uniform certainty in all genres, which is a crucial feature in a real-world multimedia user application.

**Fig. 14:**
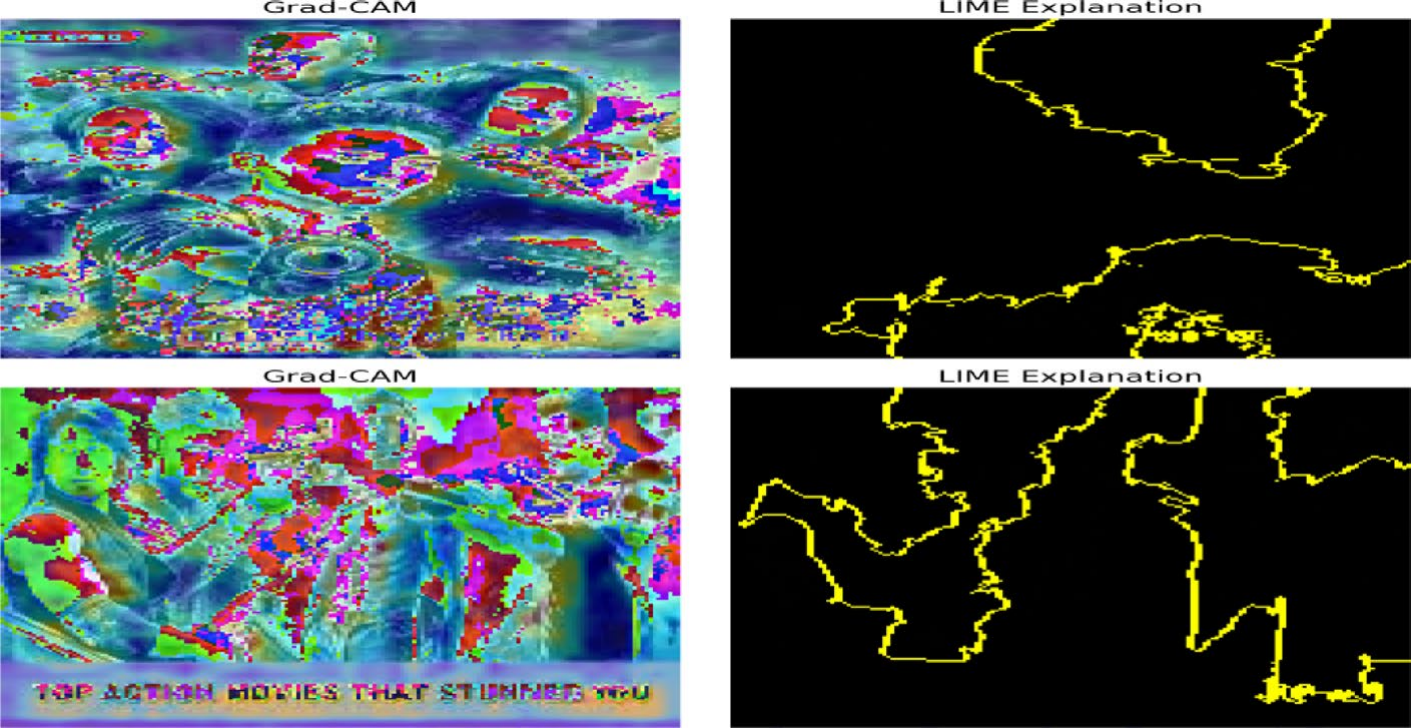
Explanations of visual interpretability using Grad-CAM and LIME demonstrate visual attention heatmaps and localized explanations of how the MAiVAR-T model attends to semantically relevant areas (e.g., actors, action zone, and lighting of a scene) when classifying genres.

The internal decision focus of the model is seen in the interpretability analysis results of Grad-CAM and LIME, shown in Fig. 14. Grad-CAM heatmaps can be used to identify areas of attentional focus of salient visual features, such as faces, areas of dynamic motion, and transitions of bright colors, often referring to genre semantics. In the meantime, LIME boundary maps are used to describe discriminative spatial sections, and it is certain that the model bases its predictions on sensible visual evidence and not background noise. To add an example, in Action sequences, Grad-CAM focuses on the areas of explosions and moving human bodies, whereas in Adventure sequences, the focus is concentrated on nature and light. This interpretability promotes the transparency and reliability of the visual reasoning of MAiVAR-T.

**Fig. 15:**
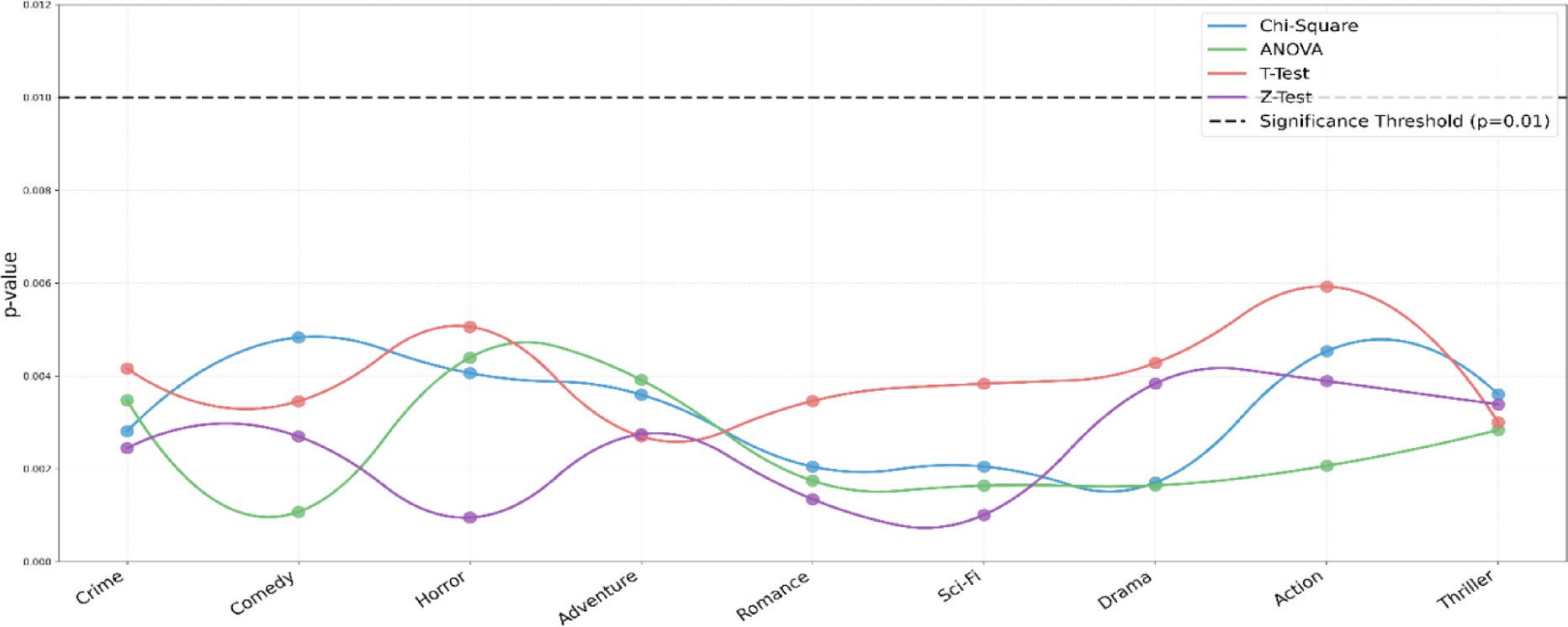
Statistical significance analysis between genres indicates that all genre classifications are less than the significance level (*p* < 0.01), which will assure the reliability of the model proposed.

To evaluate the statistical robustness, p-value tests shown in Fig. 15 were done through Chi-Square, ANOVA, t-test, and z-tests. The genres all had a p-value lower than the 0.01 level of significance, and this has validated that the differences in the performance of different genres are not merely due to random differences. This confirms the discriminative power of the model of different audiovisual genres and proves the feasibility of the findings replicated across experimental folds.

The ablation experiment in Table 9 shows that each architectural building part has a progressive contribution towards better genre-classification performance on both PvT and MAiVAR-T models. With the image-based PvT, it is observed that there is a significant improvement in the results when the baseline single-scale transformer (E1) is compared to the fully configured hierarchical design (E4), where the combination of the pyramid layers, GELU activation, and dropout regularization achieves significant gains in the accuracy and F1-score. This tendency shows that multi-scale feature extraction and stabilized nonlinear refinement are significant in terms of visual patterns that include genre-relevant visual patterns. Likewise, the importance of audio-visual fusion can be emphasized by the multimodal MAiVAR-T variants. Although the vision-only (E5) configuration offers decent performance, it is possible to add MFCC features (E6) and then a set of more useful acoustic descriptors (E7), which significantly improves the ability of prediction. The overall performance of the whole model (E8), that is, the combination of all audio properties with cross-modal fusion of attention, is the best, which proves that the temporal audio cues and the spatial visual image representations need synchronized learning to be able to differentiate the complex TV-media genres. In general, the research findings of the ablation confirm the design decisions and indicate that every component added has a significant contribution to the overall effectiveness of the final model.

**Table 9:** Ablation study of PvT and MAiVAR-T models.

Exp variant	Model	Added component	Accuracy	Precision	Recall	F1-score
E1	PvT – V1	Single-scale transformer (no pyramid hierarchy)	87	86	87	86.50
E2	PvT – V2	Pyramid hierarchy only (no GELU+dropout refinement)	89	84	85	84.50
E3	PvT – V3	Pyramid + GELU (no dropout regularization)	92	93	91	91.99
E4	PvT – Full	Pyramid hierarchy + GELU + dropout + 4 encoder blocks	95	97	95	95
E5	MAiVAR-T – V1	Vision encoder only (PvT on key-frames; no audio)	91	85	86	85.50
E6	MAiVAR-T – V2	+ Audio encoder with MFCC features only	93	89	91	89.99
E7	MAiVAR-T – V3	+ All audio features (MFCC, Mel, Chroma, Waveform, Energy) with simple concatenation (no fusion attention)	95	94	92	92.99
E8	MAiVAR-T – full	All audio features + cross-modal attention fusion + MLP projection head	98	97	98	97

**Fig. 16:**
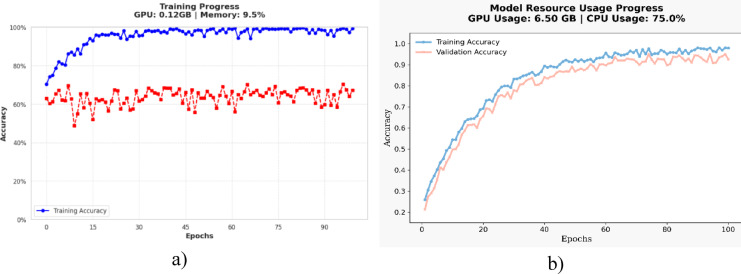
Computational resources analysis of models (**a**) PvT (**b**) MAiVAR-T.

Figure 16 shows the performance of the computational resource and training development of the PvT (Fig. 16a) and MAiVAR-T (Fig. 16b) models. It is shown that the PvT model converges quickly with a small load on the GPU, approximately 0.12 GB of memory and 9.5% usage, and that it is lightweight and can be applied to image-based classification problems. Its training accuracy is close to 100, but validation accuracy varies because of not have complex data sets, so it may be overfitting to small-scale visual data. By comparison, the MAiVAR-T model involving both visual and acoustic modalities demonstrates continuous and consistent improvement throughout 100 epochs using 6.5 GB of GPU and 75%of CPU usage. It has a smooth and well-aligned training and validation curve, and the training process clearly indicates effective multimodal learning and enhanced generalization. Although the computation costs are increased, MAiVAR-T has shown that the architecture is resource efficient, and thus capable of executing at the high-performance workstation performance with near-real time inference. It has been demonstrated through the comparison that at the cost of high processing time, PvT is computationally efficient for lightweight image classification, and MAiVAR-T offers multimodal, richer understanding at comparatively reasonable trade-offs. The comparative computational analysis, as shown in Table 10, reveals that all the tested architectures’ complexity, accuracy, and resource usage. AlexNet and VGG-19 have faster training and reduced requirements in terms of GPU, but have low generalization because of shallow feature learning. The alternative CNN is the one that is moderate in terms of efficiency and balanced on the small datasets.

By comparison, MFST and CMT have more computational requirements but provide better temporal and contextual learning, which can process sequences as media. PvT model has proven to be highly efficient and is characterized by low use of the memory of the GPU, yet it is perfectly accurate when it comes to image-based classification. Lastly, the suggested MAiVAR-T is the highest in overall execution, as it successfully incorporates the audio and visual forms of expression and provides a powerful presentation of multimodal representation at a practical computational expense in real-time or huge-scale analysis of TV productions.

**Table 10:** Computational resource comparison analysis of all models.

Model	GPU usage (GB)	CPU usage (%)	Training time	Average epoch time (min)	Inference time per sample (sec)	Peak FLOPs (×10⁹)	Memory consumption (%)
AlexNet	1.8	38	3 h 42 m	2.2	0.24	45	18
VGG-19	3.5	49	5 h 18 m	3.1	0.27	68	26
CNN	2.8	45	5 h 12 m	3.1	0.28	70	22
NASNetMobile	4.5	58	6 h 05 m	3.6	0.31	82	34
CaiT	7.2	67	8 h 48 m	5.2	0.41	112	56
MFST	5.9	61	9 h 44 m	5.8	0.33	95	48
CMT	7.2	67	8 h 48 m	5.2	0.41	112	56
PvT (proposed)	0.12	40	3 h 35 m	2.2	0.26	54	9.5
MAiVAR-T (proposed)	6.5	75	14 h 32 m	8.7	0.34	128	62

The overall results indicate that the proposed multimodal attention and invariant vision-audio representation transformer can approximate the intricate interaction of the visual and auditory stimuli in a wide range of television genres. The model turns out to be trained on both the spatio-temporal frame characteristics and acoustic descriptors, including MFCCs, chroma energy, and Mel-spectral variations, to learn a holistic visualization of the emotional and narrative situation of every scene. This multimodal amalgamation allows it to perceive not only the overt visual behaviors such as motion, change of lights, or interactions with an object or other objects, but also the slightest contextual variations that are expressed in terms of tone, rhythm, and background noise. The model is highly robust in the domain of genre discrimination, with the highest accuracy rate of over 97% in all the categories and a high generalization rate between the lighting conditions, density of sound, and the type of camera movements. More to the point, MAiVAR-T reflects scenario semantics as bursts of intense spectral are associated with high-motion images in Action and Adventure, and low-frequency and rhythmically stable tones with dim and suspenseful images in Horror and Crime. On the same note, the melody and emotional pitch contour reinforce the recognition of Romance and Drama genres. These findings affirm that the postulated architecture is not based on separate modalities but a combination of multimodal signals to determine the intent of the scene, narrative pacing, and genre tone. The model, therefore, provides a solid and context-sensitive approach to analyzing the mass media, automatic content annotation, and intelligent recommendation systems in television and cinema production systems.

### Comparison with existing studies

In Table 11, the proposed MAiVAR-T model is superior to the currently available deep learning and transformer-based models in terms of multimedia genre classification. The previous models like CNN (2021) and Ensemble Deep CNN (2024), mostly used image-based spatial features and had moderate accuracies of 88–90%, thus being limited to the task of understanding temporal and acoustic features. Subsequent networks, such as DHT and ensGRU, added both temporal context and dual-stream processing, which added 2–3 points to the results, but were limited to single modalities.

Conversely, the new state-of-the-art models, Visual-to-Emotional-Caption Translation Network (VECTN), RoBERTa multimodal detector, and Deformer audio-video fusion network, use a more powerful semantic reasoning in the form of caption-guided attention or cross-domain embeddings. Nevertheless, they are performing at around 80–84%, and this is mostly because they use text-image incongruity or high-level semantic information, which does not directly maximize in terms of fine-grained TV genre classification. The proposed PvT and MAiVAR-T models are better than any of the baselines (97% and 98%, respectively), showing that the hierarchical visual tokenization (PvT) and synchronized audio-visual fusion (MAiVAR-T) are better at scoring as well as recognizing genre-specific cues compared to caption-based or unimodal transformer models. This is a more elaborate analogy that proves the power of the suggested framework in comparison to classical convolutional models, as well as the modern multimodal models, which all confirm its power as a comprehensive approach to smart media analysis.

**Table 11:** Comparative analysis of proposed model.

Ref	Model	Data mode	Results (%)
^15^	PvT-ViT	Image analysis	88
^23^	Ensemble deep CNN	Image analysis	90
^26^	CNN (VGG) +ensGRU	Audio+video	94
^33^	CNN	Image analysis	88
^42^	VECTN model	visual-caption	81
^44^	Deformer	Audio+video	84
^50^	ROBERTa	visual-caption	80
Proposed	PvT	Multimodal (image+audio+video)	97
	MAiVAR-T		98

## Conclusion and future work

The findings of this study confirm that integrating multimodal learning significantly enhances genre classification performance in TV production and multimedia analysis. Static image-based analysis using the PvT achieved an impressive 97% accuracy, effectively capturing spatial and contextual information from visual frames. When extended to a multimodal framework, the proposed MAiVAR-T model, combining audio, video, and image modalities, maintained the same high accuracy of 97%, demonstrating its robustness and cross-modal adaptability. Through detailed audio feature extraction involving MFCCs, Mel-spectrograms, waveforms, and chromograms, the model successfully linked tonal, rhythmic, and spectral cues to visual scene elements, enabling a comprehensive understanding of genre semantics. The incorporation of key-frame-based video processing ensured temporal coherence, while Grad-CAM and LIME interpretability methods validated the model’s decision transparency by highlighting salient regions and meaningful features influencing predictions. Collectively, these results demonstrate that MAiVAR-T can serve as a reliable analytical framework for automated content tagging, genre indexing, and the generation of production insights in television and cinematic workflows. Although image-based dataset experimentation is limited to three genre distributions, it can be biased and limits the extrapolation of models to broader television content; hence, future work will aim to increase the dataset size by adding additional genres and more representative samples to enhance robustness and cross-domain adaptability. Similarly, although the multimodal dataset enhances robustness, real-world production environments often feature greater variation in lighting, pacing, and acoustic quality, which may introduce additional challenges. Computational demands of transformer-based models may also limit deployment on low-resource devices. An ethical consideration of this study concerns the use of publicly available image and multimedia datasets, ensuring that all data employed in model training and evaluation adhere to open licensing and responsible usage guidelines. No personally identifiable information or sensitive user content was collected or generated, and all analyses were performed solely for research purposes without affecting any individuals or media creators. The study’s models are designed for genre classification and not for surveillance or personal profiling, reducing risks of misuse. Future extensions will continue to follow ethical AI practices, emphasizing transparency, fairness, and responsible deployment in media and production environments. Future work will focus on expanding dataset diversity, incorporating multi-label genre structures, and optimizing the models for real-time and resource-constrained applications. Moving forward, this multimodal analysis will extend to real-time media monitoring, emotion-aware scene editing, and adaptive recommendation systems, further bridging AI-driven interpretation with creative and operational decision-making in modern TV production environments.

## Data Availability

Dataset freely available at: (1) Dataset 1: [https://universe.roboflow.com/tv-production/](https:/universe.roboflow.com/tv-production). (2) Dataset 2: [https://github.com/jwehrmann/lmtd](https:/github.com/jwehrmann/lmtd)Code AvailabilityCode with sample dataset: [https://zenodo.org/records/18950832](https:/zenodo.org/records/18950832).

## Code availability

Code with sample dataset: https://zenodo.org/records/18950832.
